# Clinical performance of long-term temporary fixed dental prostheses fabricated from CAD/CAM resin-based composite

**DOI:** 10.1007/s00784-025-06259-8

**Published:** 2025-03-12

**Authors:** Florian Dold, Karolina Mueller, Martin Butz, Sebastian Hahnel

**Affiliations:** 1 Department of Prosthetic Dentistry, University Hospital Regensburg, Regensburg, Germany; 2 Center for Clinical Studies, University Hospital Regensburg, Regensburg, Germany; 3 Private Dental Practice, München, Germany

**Keywords:** Fixed interim restoration, Composite, Crown, Fixed dental prostheses

## Abstract

**Objectives:**

To determine the clinical performance of long-term temporary fixed dental prostheses (LTFDPs) manufactured from CAD/CAM temporary resin-based composite.

**Materials and methods:**

Retrospective data of 46 patients supplied with 73 LTFDPs (partial coverage crowns, crowns, fixed dental prostheses) manufactured from a CAD/CAM resin-based composite luted either temporarily, selfadhsesively, or adhesively were analyzed for failures and complications.

**Results:**

Datasets of 44 patients with 71 LTFDPs (12 partial-coverage crowns, 31 crowns, 28 fixed dental prostheses) were included in the analyses; median observation time was 362.0 days. Failures due to fracture occurred in 14.1% and decementation was observed in 18.1% of all LTFDPs. There was no statistically significant difference in failure rates between partial coverage crowns, crowns, and fixed dental prostheses. The probability of decementations was significantly higher in restorations that had been luted temporarily or selfadhesively than in those that had been luted adhesively.

**Conclusions:**

LTFDPs fabricated from resin-based temporary composite with low filler content feature relevant failure rates, which is independent on the type of restoration. Decementation is a frequently observed complication and dependent on the mode of luting.

**Clinical Relevance:**

LTFDPs fabricated from resin-based composite have similar failure rates as reported for polymethyl methacrylate based LTFPDs. Adhesive luting might help to reduce the probability of decementation.

## Introduction

In fixed prosthetic dentistry, a well-crafted temporary restoration is an important and integral part in the process of providing a successful indirect restoration [1]. Temporary restorations protect the prepared teeth from mechanical forces and thermal and chemical irritants. They shape marginal gingival areas, and preserve or restore masticatory functionality, phonetics as well as aesthetics until insertin of the final prosthesis [2]. Temporary restorations are also valuable tools for diagnostic purposes as they help to develop and evaluate functional, occlusal, and aesthetic parameters in order to identify the optimal treatment outcome before beginning or completing definitive treatment procedures [3, 4]. As the conditition of the abutment teeth is a highly relevant issue in prosthetic dentistry as it directly affects the performance and longevity of the restoration itself [5], it can be necessary to continuously treat or monitor potential abutment teeth with questionable prognosis over a longer period of time. In these situations, long-term temporary fixed dental prostheses (LTFDPs) may be used during periodontal therapy or continuous risk assessment.

Temporary FDPs can either be manufactured directly in a chair- or labside process or indirectly using CAD/CAM technology. In most cases, temporary FDPs produced with direct techniques are used for shorter periods such as weeks or months, whilst temporary FDPs fabricated with indirect techniques may be used for a period of up to three years, e.g. in LTFDPs [6, 7]. Most directly produced temporary FDPs are fabricated using the overimpression technique and autopolymerizing resin-based composites and may feature some drawbacks resulting from suboptimal conditions during the manufacturing process. Voids or contamination from the oral cavity may cause material inhomogeneities that can foster discoloration and impair mechanical strength and fit. Polymerization shrinkage may also negatively affect the fit and marginal adaptation of the temporary FDPs. Many of these issues can be overcome with CAD/CAM technologies using industrially manufactured polymer blocks or discs. Laboratory studies reported that FDPs milled from CAD/CAM acrylate-based materials featured higher strength and better marginal accuracy than FDPs fabricated directly with conventional resin-based composite materials, especially after artificial aging [8]. These CAD/CAM materials benefit from optimized and standardized industrial polymerization conditions, which occur at higher temperatures and pressure, resulting in a higher degree of polymerization compared to directly fabricated resin-based materials. Consequently, CAD/CAM resin-based materials exhibit improved mechanical properties, enhanced biocompatibility, and reduced biofilm formation compared to directly processed resin-based alternatives [9–14]. In addition to that, the materials caused less antagonist wear in chewing simulations [15]. While laboratory studies conclude that these materials might suitable for application in LTFDPs [15, 16], no data on the clinical performance of these materials are currently available.

Against this background, the current study retrospectively analyzed the clinical performance of LTFDPs fabricated from novel CAD/CAM resin-based composites with little filler content. It was hypothesized that (1) the frequency of failures due to fracture is independent of the type of restoration and (2) that the frequency of decementations is independent of the type of the luting agent.

## Method and materials

Patients of the Department of Prosthetic Dentistry at the Regensburg University Medical Center who received prosthetic treatment with long-term temporary fixed dental prostheses (LTFDPs) between April 2022 and Januar 2024 were included in the retrospective analysis. The study was designed in accordance with the STROBE checklist for reporting observational studies. Only datasets of patients supplied with LTFDPs– including partial-coverage crowns, crowns, and terminal fixed dental prostheses– fabricated from a CAD/CAM resin-based composite with 27% filler content (Structur CAD, VOCO GmbH, Cuxhaven, Germany) were included in the retrospective analyses. Suitable patients were identified by screening the institutional electronic database for appropriate fee scale items and additional manual screening of the retrieved clinical datasets for eligibility.

**Fig. 1:**
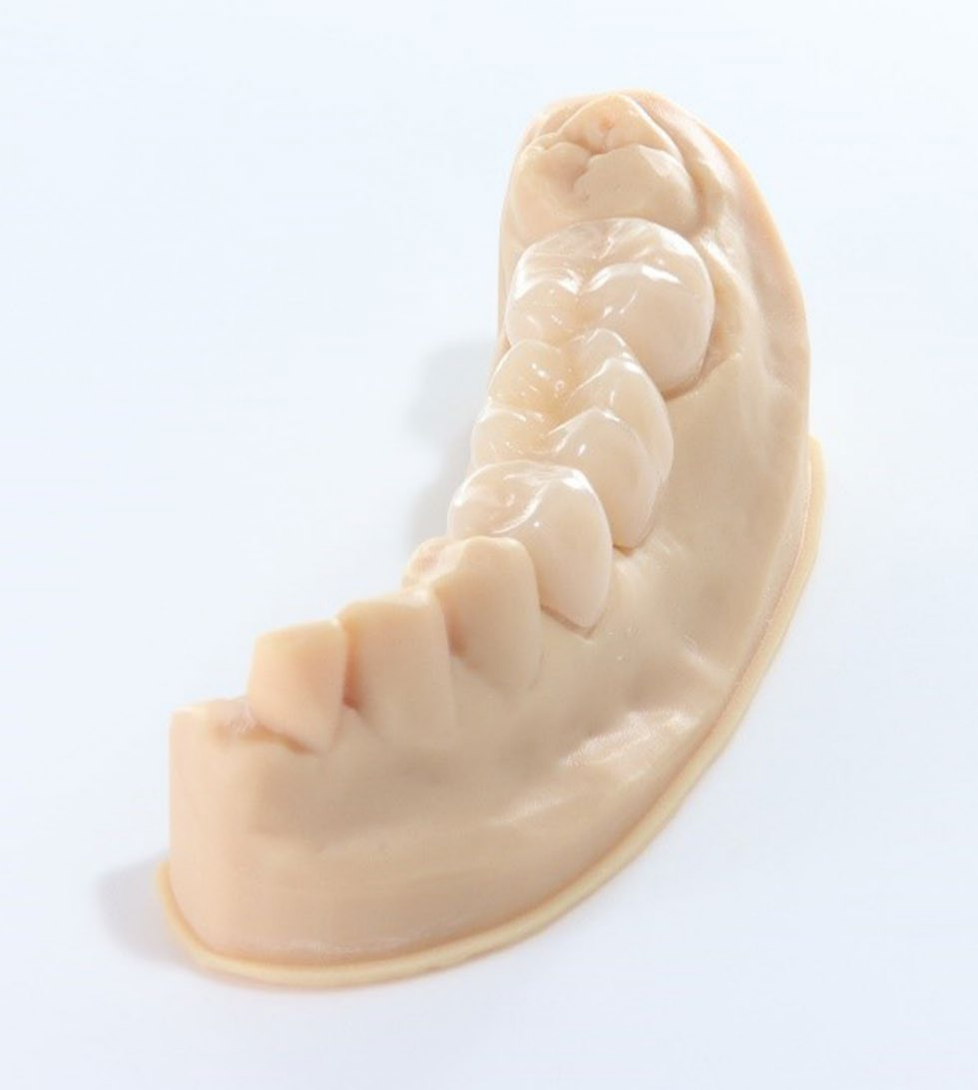
Sample of a three-unit fixed dental prostheses fabricated from a CAD/CAM resin-based composite with 27% filler content (Structur CAD)

Patients had been treated by either experienced dentists specialized or currently specializing in prosthodontics or supervised undergraduate students (who had successfully completed their preclinical education). Tooth preparation was performed in accordance with the commonly accepted preparation designs for partial restorations (defect-oriented and non-retentive, rounded edges and internal transitions) and fixed dental prostheses fabricated from tooth-coloured materials. Each LTFDP was manufactured at the dental laboratory at the Department of Prosthetic Dentistry at Regensburg University Medical Center using datasets gathered from digitalized gypsum models or digital intraoral impressions. All LTFDPs were designed with the same dental CAD software (InLab CAD, Dentsply Sirona, Bensheim, Germany) and milled dry from standard blanks using a five-axis dental milling machine (InLab MCX5, Dentsply Sirona) equipped with burs for composite and set in composite mode. The guidelines issued by the manufacturer of the CAD/CAM resin-based composite regarding minimal cervical wall thickness (single crowns– 0.6–0.8 mm; anterior fixed dental prostheses 0,8 mm; posterior fixed dental prostheses 1.0 mm), minimal occlusal wall thickness (1.2–1.5 mm), and maximum number of adjacent pontics in fixed dental prostheses (2) were carefully followed. After milling, try-in and occlusal adjustments with fine diamond burs, LTFDPs were polished in the dental laboratory with goat hair brushes and paste (Fig. 1).

After cleaning the abutment teeth and implants with pumice slurry and careful air-abrasion of the inner surface of the LTFDPs with aluminium oxide (0.1–0.2 MPa, 50–100 μm), the LTFDPs where luted either with an eugenol-free temporary zinc oxide-based cement (Tempbond NE, Kerr), a self-adhesive luting cement (RelyX Unicem 2 Automix, Solventum, St. Paul, USA), or a resin-based composite adhesive luting material (Bifix QM, VOCO) in combination with additional bonding agents for tooth surface (Futurabond U, VOCO) and restoration (Ceramic Bond, VOCO).

The frequency of failure due to fracture of the LTFDPs as well as complications such as decementation or secondary caries of the abutment tooth were analyzed retrospectively from clinic records. Included risk factors were the type of the restoration (partial-coverage crowns, crowns, fixed dental prosthesis) and the luting agent (temporary cement, selfadhesive cement, adhesive resin-based luting composite) employed.

Median observation time (95% CI) as well as frequency of failure due to fracture and complications such as decementations and secondary caries were calculated using statistical software (SPSS 29.0.1, IBM, Armonk). Mixed effects Cox regression analysis for time until failure was performed to evaluate a dependency on the type of restoration (R 4.4.1, R for Windows, https://cran.r-project.org/) and generalized estimating equation model (GEE) with binary regression was employed to evaluate differences in decementation probability in dependence on the luting agent (SPSS). The level of significance (α) was set to 0.05.

## Results

An overall total of 73 LTFDP datasets in 46 patients were retrieved and manually screened for eligibility. Datasets of two LTFDPs in two patients were excluded as they documented a hybrid abutment LTFDP (one dataset) and an extended LTFDP fabricated outside the spectrum of indications of the restorative material (second dataset). Every LTFDP was included as a single dataset in patients who received multiple LTFDPs.

In total, datasets of 44 patients (*N* = 17/38.6% female) with 71 LTFDPs were evaluated, including partial-coverage crowns (*N* = 12/16.9%), crowns (*N* = 31/43.7%), and FDPs (*N* = 28/39.4%) on natural abutment teeth (*N* = 70) or implant abutments (*N* = 1). 51 LTFDPs (71.8%) were inserted by supervised undergraduate students. Median observation time for all restorations was 362.0 days (95% CI lower 335,9 days/upper 388.1 days). LTFDPs were either luted with temporary cement (*N* = 4, 5.6% (N partial-coverage crown/N crown/N fixed dental prostheses = 0/2/2), self-adhesively (*N* = 25, 35.2% (12/5/8)), or adhesively (*N* = 42, 59.2% (0/24/18).

10 (14.1%) LTFDPs failed during the observation time due to fracture (partial-coverage crowns: *N* = 1 (8.3%); crowns: *N* = 3 (9.7%); fixed dental prostheses: *N* = 6 (21.4%). 2/6 FDPs with two adjacent pontics failed (33.3%). Decementation was the most frequent complication and occurred in 18.1% (*N* = 13) of all LTFPDs, including 9 partial-coverage restorations (75.0%), 1 crown (3.2%), and 3 FDPs (10.7%). All decemented partial-coverage restorations were luted self-adhesively. 2/3 decemented FDPs were luted with temporary cement and 1/3 decemented FDPs as well as the decemented crown were luted adhesively. Secondary caries was identified as complication in one case (1.4%; self-adhesively luted partial coverage crown).

**Fig. 2:**
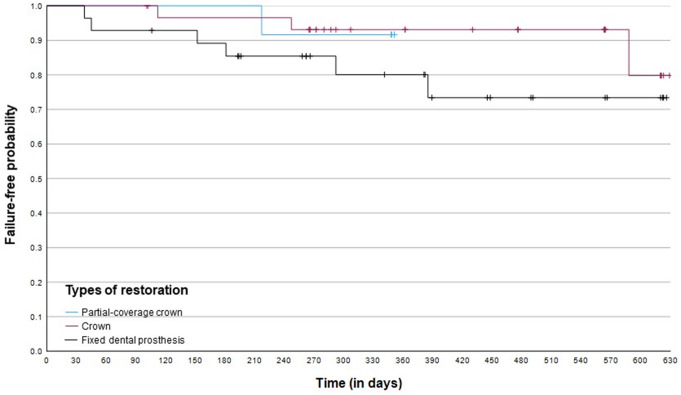
Kaplan-Meier curve for failure rate of the various types of restoration. Kaplan-Meier curve represents each restoration/tooth

As in some patients, more than one LTFDP was included in the analyses, cluster patients were taken into statistical consideration. Mixed effects Cox regression analysis for time until failure indicated no significant difference between the various types of restoration regarding time until failure, taking partial coverage crowns as reference (Table 1; Fig. 2). Odds rates for decementations were significantly higher in restorations that had been luted temporarily and self-adhesively rather than adhesively (reference) (Table 2).

**Table 1:** Mixed effects Cox regression analysis for time until failure. Reference was partial-coverage crowns. Between crown and fixed dental prothesis no significant differences were identified (HR = 0.96, 95% CI = 0.10, 1.57, *p* = 0.184)

	HR	95%CI		*p*
Crown	0.96	0.09	9.57	0.972
Fixed dental prosthesis	2.46	0.29	21.02	0.410

**Table 2:** Odds rates for decementations. Reference was adhesively luted. Between temporarely and selfadhesively luted restaurations no significant difference were identified (OR = 0.60, 95% Wald CI = 0.03, 11.32, *p* = 0.733)

Parameter	OR	95% Wald Confidence Interval		
		Lower	Upper	Sig.
(Intercept)	0.05	0.01	0.20	< 0.001
Temporarily luted	20.50	1.26	332.63	0.034
Selfadhesively luted	12.30	1.36	111.48	0.026

## Discussion

Data on the clinical performance of LTFDPs are still scarce, although achievements in CAD/CAM technologies have made this treatment option feasible by substantially decreasing production costs. Hüttig and co-workers reported a survival rate of 90.4% in conventional terminal-supported 3- or 4-unit FDPs fabricated from CAD/CAM-processed PMMA after a median observation time of 16 months [17]. In the current study, a failure rate of 14.1% after almost one year of clinical service was observed for the various restorations, which is in a similar range and comparable to temporary FDPs fabricated from temporay resin-based composite in a direct approach, which featured failure rates of 13% after a relevantly shorter service period of 45 days [18]. Nevertheless, LTFDPs have a higher failure rate in comparison to permanent all-ceramic FDPs, which range between 89.4 and 95.9% for tooth-supported crowns and fixed dental prostheses after five years [19].

No significant differences in failure rates were identified between partial-coverage crowns, crowns, and FDPs, which is why the first study hypothesis can be accepted. Similar results have been previously reported for temporary FDPs processed in a direct approach with temporary resin-based composite materials [18]. Laboratory studies have also pointed out that the CAD/CAM resin-based composite used for the fabrication of LTFDPs in the current study is suitable for application in the 3-unit FDPs for a period of up to two years [15]. The data of this clinical study, which included even 5- and 6-unit FDPs (*n* = 4), support these laboratory results. However, for the few FDPs with neighbouring pontics, a failure rate of 33.3% was identified, which indicates that these constructions might be used with with caution, although the data have to be corroborated with further clinical and laboratory data. In addition to that, compromised abutment teeth, e.g. with increased tooth mobility, might have been included in the analysis, which might particularly affect the performance of FDPs by high torsional stress. It is one of the weaknesses of the current study that the periodontal status of the abutment teeth has not been documented in a standardized and calibrated manner, which is why it has not been included as a confounder in the analyses. It is also clear that the different expertise of the clinical operators (supervised undergraduate students vs. (experienced) dentists), the distinct clinical settings with different types of restorations, and different cementation techniques might bias the results of the current study, although they represent a potential spectrum of clinical settings. Other shortcomings may include the limited observation time and the limited number of restorations as well as the retrospective approach and a potential bias by including more than one restoration for some patients in the analyses, which has, however, been addressed in the statistical analyses. In addition to that, it has been verified in the dental laboratory that the restorations meet the minimally required thickness threshold, yet the thickness has not been controlled prior insertion, which means that occlusal adjustments during try-in of the restorations may have caused a violation of the thickness threshold. Nevertheless, the current study is the first study reporting on the performance of LTFDPs fabricated from CAD/CAM resin-based composite and adds to the scientific evidence available for the performance of LTFDPs.

While failure rates of partial-coverage crowns, crowns, and FDPs were similar, decementations were most frequently observed for partial-coverage crowns. Although higher decementation rates for partial-coverage crowns had been expected as a result of non-retentive preparations, the high number of decementions was surprising, particularly as all decemented partial-coverage crowns had been luted with selfadhesive cement. Clinical studies have highlighted that tooth-colored partial coverage restorations may be successfully luted with selfadhesive cement, reporting a complication-free rate of 86% in terms of decementation after a observation period of five years in comparison to 100% in partical coverage restorations luted adhesively with resin-based composite [20]. These observations underline that luting procotols for partial-coverage crowns fabricated from CAD/CAM resin-based composite should be be investigated in more detail in future studies, although it is clear that the exclusive use of selfadhesive cement for luting partial-coverage crowns might bias the outcome of the current study. If expected that the LTFDP is going to be in service for more than 30 days, the manufacturer recommends adhesive luting rather than cementing with temporary cements. In those restorations (only crowns and FDPs) that had been adhesively luted, a decementation rate of 4.8% was observed. In fact, statistical analysis revealed that the probability of decementation was significantly higher in restorations that had been luted temporarily and self-adhesively rather in comparison to those that had been luted adhesively. These observations suggest rejection of the second research hypothesis and indicate that LTFDPs that are intended to be in clinical service for extended periods should be cemented adhesively with resin-based composites. Due to the retrospective character of the study, it was not possible to analyze the debonding pattern, which might have added some relevant information on the interaction between the resin-based composite and the luting material.

## Conclusion

LTFDPs fabricated from resin-based composite feature clinically sufficient to good failure rates, which are comparable to data reported in the literature for LTFPDs manufactured from polymethyl methacrylate. Luting LTFDPs adhesively with resin-based composites might help to reduce the probability of decementations in restorations with higher expected service time.

## Data Availability

No datasets were generated or analysed during the current study.
